# Postprandial Cardiometabolic Parameters in Older Adults with Normal-Weight Obesity: A Cross-Sectional Pilot Study

**DOI:** 10.3390/metabo15080550

**Published:** 2025-08-15

**Authors:** Dhanya O. Pathangi, Alexis R. Quirk, Jenna K. Schifferer, Sarah E. Fruit, Morgan E. Higgins, Emily R. Wolf, Cindy E. Tsotsoros, Sam R. Emerson, Bryant H. Keirns

**Affiliations:** 1 Department of Nutrition and Health Science, Ball State University, Muncie, IN 47303, USA; 2 Department of Human Development and Family Science, University of Rhode Island, Kingston, RI 02881, USA; 3 Department of Nutritional Sciences, Oklahoma State University, Stillwater, OK 74075, USA; 4 545 Health Professions Building, Ball State University, Muncie, IN 47306, USA

**Keywords:** aging, body fat percent, postprandial triglycerides, inflammation, intestinal permeability

## Abstract

Background/Objectives: Normal-weight obesity describes those with a normal body mass index (BMI) and high body fat percent. Older adults with normal-weight obesity (NWO-O) are at increased risk for cardiovascular disease (CVD), but underlying mechanisms remain unclear. This pilot study examined whether NWO-O had an unfavorable cardiometabolic response to acute high-fat meal intake compared to normal BMI, low body fat percent individuals that were both older (NWL-O) and younger (NWL-Y). Methods: Participants (N = 29) with a normal BMI were grouped as follows: NWL-Y (18–35 years, low body fat percent; n = 12), NWL-O (≥60 years, low body fat percent; n = 9), and NWO-O (≥60 years, high body fat percent; n = 8). All participants completed an abbreviated fat tolerance test (75 g fat). Fasting and 4 h blood samples were collected to measure lipids (triglycerides and high-density lipoprotein cholesterol [HDL-C]), biomarkers of intestinal permeability (lipopolysaccharide binding protein [LBP] and soluble cluster of differentiation [sCD14]), and the inflammatory marker interleukin (IL)-6. Results: NWO-O had higher percent, absolute, and trunk fat compared to NWL-Y and NWL-O (*p*’s ≤ 0.01). Conversely, percent lean mass was lower in NWO-O versus both NWL groups (*p*’s ≤ 0.01). NWO-O had higher fasting triglycerides than NWL-Y (*p* < 0.05), but all groups were in the clinically normal range on average (≤107 mg/dL). However, NWO-O had higher 4 h triglycerides (239.4 ± 101.0 mg/dL) compared to NWL-Y and NWL-O (*p* < 0.01), consistent with an adverse response. The absolute change in triglycerides was higher in NWO-O relative to NWL-Y (*p* < 0.01), but not compared to NWL-O (*p* = 0.06). Fasting IL-6 was higher in NWO-O relative to NWL-Y (*p* < 0.05). Fasting and 4 h sCD14 were similarly higher in NWL-O and NWO-O versus NWL-Y (*p*’s < 0.01). Conclusions: NWO-O had an exaggerated postprandial triglyceride response compared to younger and similar-aged NWL individuals, which could reflect hepatic very low-density lipoprotein overproduction or impaired triglyceride clearance. Future work should continue to investigate the role of postprandial dyslipidemia in NWO-O’s reported CVD risk.

## 1. Introduction

Cardiovascular disease (CVD) remains the leading cause of death in the United States [1]. Obesity, traditionally defined as a body mass index (BMI) ≥ 30.0 kg/m^2^, is an established risk factor for CVD [2]. However, it is now understood that high relative body fat is related to CVD risk regardless of BMI classification [3]. As one example, adults with normal-weight obesity (NWO)—or those with a normal BMI and high body fat percent—appear to be at increased cardiometabolic risk [3,4]. Indeed, adults with NWO display a number of CVD risk factors (e.g., low-grade inflammation, oxidative stress, low cardiorespiratory fitness) and are at 2.2× increased risk for CVD relative to normal BMI, low body fat percent counterparts (i.e., normal-weight lean individuals or NWL) [3,5,6].

While the CVD risk associated with NWO in general adult populations is increasingly appreciated, less work has focused on older adults with NWO (NWO-O). Notably, ~67% of adults in the United States ≥ 60 years that maintain a normal BMI are considered NWO (~14 million total) [7,8,9]. Much like younger populations, NWO is associated with increased risk for CVD mortality in both males and females over 60 years [10]. Despite recognition that NWO-O are at heightened CVD risk, studies suggest their clinical presentation may be unremarkable based on typical assessments (e.g., normal BMI, blood pressure, or select cholesterol metrics) [3,10,11]. Further, relying on BMI in this population may fail to identify unfavorable fat distribution patterns (i.e., higher visceral fat and/or lower subcutaneous fat) and/or low lean body mass. Taken together, these factors increase the likelihood that CVD risk will be underestimated in this population. Finally, studies to date in NWO-O have largely been population-based in nature, focusing on CVD outcomes, prevalence, and physical function [7,10,12,13]. As a result, experimental studies examining potential mechanisms driving CVD risk in NWO-O are warranted.

High-fat meals consumed in Westernized cultures induce adverse changes in cardiometabolic factors that (1) may not be apparent under fasting conditions and (2) may promote CVD if consumed on a chronic basis. For example, an exaggerated postprandial triglyceride response to acute high-fat meal consumption is an independent CVD risk factor and can occur in individuals with normal fasting lipids [14,15]. Physiologically, this response is due in part to dietary fat absorption and transport in chylomicrons, followed by subsequent competition between chylomicron-triglycerides and very low-density lipoprotein (VLDL)-triglycerides. The resulting large postprandial triglyceride excursions may further promote CVD by decreasing high-density lipoprotein cholesterol (HDL-C) in a cholesteryl ester transfer protein (CETP)-dependent manner [15]. Further, high-fat meals are known to disrupt the intestinal barrier, leading to translocation of bacterial components (e.g., lipopolysaccharide or LPS) into the circulation [16,17]. This low-grade endotoxemia is associated with increased secondary biomarkers of LPS exposure, including LPS binding protein (LBP) and soluble cluster of differentiation 14 (sCD14) alongside evidence of inflammation (e.g., increased interleukin [IL]-6) [16]. Taken together, consumption of high-fat meals can negatively impact a host of cardiometabolic parameters and may also reveal undetected CVD risk.

Due to the limited knowledge on mechanisms driving CVD in NWO-O, as well as their largely normal clinical profile, we aimed to characterize this population’s acute response to a high-fat meal. To accomplish this goal, we compared postprandial lipids, markers of LPS exposure/inflammation in NWO-O to older adults with NWL (NWL-O) and younger adults with NWL (NWL-Y). We hypothesized that NWO-O would display adverse changes in cardiometabolic factors post-high-fat meal relative to both lower body fat comparator groups.

## 2. Materials and Methods

### 2.1. Participants

Participants (N = 29) with a normal BMI (18.5–24.9 kg/m^2^) were recruited into one of three groups based on age (younger or older adults) and body fat percent (low or high body fat percent) for this cross-sectional pilot study: NWL-Y (n = 12), NWL-O (n = 9), and NWO-O (n = 8). NWL-Y was 18–35 years, while NWL-O and NWO-O were ≥60 years. The body fat percent cutoff for NWO was ≥33.0% (females) and ≥23.0 % (males), and NWL groups were below these thresholds as previously described [5,18]. Beyond meeting group criteria, participants were not pregnant/expecting to become pregnant (NWL-Y), free of cardiometabolic and inflammatory conditions, did not use lipid-lowering drugs, illicit drugs, or tobacco products, and did not have a pacemaker. Further, all female participants ≥ 60 years had reached postmenopausal status. All study procedures were conducted in accordance with the Declaration of Helsinki and approved by the Ball State University Institutional Review Board (IRBNET #2140256-1).

### 2.2. Initial Assessment

Participants were recruited through flyers, mass emails, and word of mouth. Interested individuals were asked to provide a self-reported BMI and answer questions related to inclusion/exclusion criteria. Those meeting initial study criteria were invited for an in-laboratory screening visit. At this screening visit, participants’ BMI was confirmed in a singlet measurement. More specifically, height was measured to the nearest 0.1 cm using a stadiometer with shoes removed (Seca). Body weight was measured (InBody 570) while participants were wearing street clothes (except shoes) to the nearest 0.1 lb. Body fat percent was estimated using a bioimpedance scale (InBody 570). Final body fat percent values were based on dual-energy X-ray absorptiometry (DXA) scans < 2 weeks later (see Body Composition Assessment). Lastly, several one-time measurements were collected (e.g., blood pressure, waist circumference). Individuals satisfying the inclusion criteria for a study group were invited back for an oral fat tolerance test.

### 2.3. Fat Tolerance Test

Prior to the fat tolerance trial, participants fasted for ≥10 h, avoided exercise and alcohol for ≥24 h, and avoided anti-inflammatory medications for ≥72 h. First, a whole blood sample was obtained from an antecubital vein by single venipuncture. Next, a high-fat meal (252 g of Marie Callender’s Chocolate Satin Pie: 75 g total fat [46 g saturated fat]; 98 g carbohydrates [69 g sugar, 6 g fiber]; 10 g protein; 1104 kcal) was consumed in ≤20 min. A 75 g fat bolus was administered based on current recommendations for oral fat tolerance testing [15,19]. After completing the high-fat meal, participants were allowed to leave the lab with restrictions (i.e., only consume water, avoid physical activity/planned exercise). This protocol has been validated against controlled, in-laboratory fat tolerance tests and used by our group previously [20]. A final blood sample was obtained 4 h after the high-fat meal was completed, as this time point corresponds with peak postprandial triglycerides on average [15,19].

### 2.4. Body Composition Assessment

Body fat percent, absolute body fat, absolute trunk fat (an indirect estimate of visceral fat), lean mass percent, and absolute lean mass were determined by full-body DXA scans (Lunar iDXA; GE HealthCare, Chicago, IL, USA). Waist circumference was measured using a Gulick tape at the level of the umbilicus by trained laboratory personnel as previously described [21].

### 2.5. Biochemical Analyses

At baseline and 4 h time points, ~12 mL of whole blood was collected. ~2–3 mL was treated with lithium heparin and used to measure lipids (i.e., triglycerides, cholesterol metrics), glucose, and liver enzymes using a clinical chemistry analyzer (Piccolo Xpress; Abbott, Chicago, IL, USA) with Lipid Panel Plus reagent disks. The remaining whole blood was transferred to serum separator tubes, allowed to clot for 25 min, and centrifuged (2500 RPM at 4 °C for 15 min) to obtain serum. Serum was then aliquoted immediately and stored at −80 °C for future analyses. Commercially available ELISAs were used to measure LBP (Ray Biotech, Peachtree Corners, GA, USA), sCD14 (R&D Systems, Minneapolis, MN, USA), and IL-6 (Abcam, high-sensitivity kit, Waltham, MA, USA). All assays were carried out in accordance with manufacturers’ instructions.

### 2.6. Physical Activity and Diet Assessments

To assess habitual diet, all participants were asked to complete a three-day food record three days immediately prior to the fat tolerance test, irrespective of weekday or weekend. Daily energy intake, macronutrients, other select nutrients (i.e., saturated fat, sodium, fiber), and healthy eating index (HEI) 2015 were then calculated and averaged using the Nutrition Data System for Research Software (NDSR 2024; University of Minnesota, Minneapolis, MN, USA). Participants also wore a fitness tracking watch (Garmin Vivosmart 5) for three days leading up to the fat tolerance test to determine average steps/day and moderate-to-vigorous physical activity.

### 2.7. Statistical Analyses

No studies to date have examined postprandial cardiometabolic factors in NWO-O to date, making an a priori power analysis challenging. For this reason, we considered this work an initial pilot study to inform future research. All data were first checked for normality using the Shapiro–Wilk test. Continuous data following the normal distribution were analyzed using one-way ANOVA. Following significant omnibus ANOVAs, Tukey’s post hoc test was employed to determine group differences between NWL-Y, NWL-O, and NWO-O. When data did not fit the normal distribution (and could not be corrected by log transformation), Kruskal–Wallis and Dunn’s test were utilized as non-parametric equivalents. Categorical data (only sex distribution and ethnicity in this report) across study groups were analyzed using the chi-squared test. Pearson correlations were calculated to assess relationships between body fat metrics and 4 h triglycerides. All data are presented as mean ± SD, and alpha was set at 0.05. 95% confidence intervals and effect sizes were calculated for main study outcomes (Supplementary Table S1). Data were analyzed and plotted in GraphPad Prism 9.0.

## 3. Results

### 3.1. Participant Characteristics

Participant characteristics can be found in Table 1. Age was similarly higher in NWL-O and NWO-O compared to NWL-Y (*p* < 0.0001), and sex distribution did not differ across groups. No between-group differences were detected for BMI or waist circumference. Waist circumference to height ratio (WHtR) was greater in NWO-O compared to NWL-Y (*p* < 0.05). NWO-O had higher percent body fat, as well as absolute body fat and trunk fat (*p*’s ≤ 0.01). Conversely, percent lean mass was lower in NWO-O compared to both NWL-Y and NWL-O (*p*’s < 0.01). Absolute lean mass was similar in NWL-O and NWO-O and lower than NWL-Y (*p* < 0.01). Fasting total-, LDL-, and non-HDL-cholesterol were greater in NWL-O and NWO-O compared to NWL-Y (*p*’s ≤ 0.01). No differences were noted for fasting glucose, ALT, and AST or systolic/diastolic blood pressure across study groups.

Total energy and carbohydrate intake were higher in NWL-Y versus NWL-O, while NWO-O was intermediate to these groups (*p*’s < 0.05). No other differences in diet-related data were observed. Average steps per day did not differ between groups. However, NWO-O recorded significantly less moderate-to-vigorous physical activity than NWL-Y, and NWL-O was similar to both of these groups (*p* < 0.05).

### 3.2. Fasting and Postprandial Triglycerides and HDL-C

**Figure 1:**
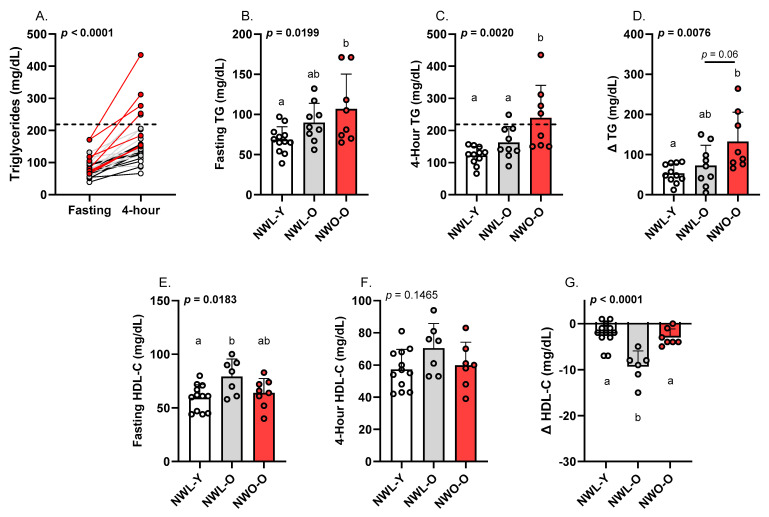
Fasting and postprandial triglycerides and HDL-C: (**A**) individual triglyceride responses, (**B**) fasting triglycerides, (**C**) 4 h triglycerides, (**D**) absolute change (∆) in triglycerides from baseline, (**E**) fasting HDL-C, (**F**) 4 h HDL-C, (**G**) absolute change (∆) in HDL-C from baseline. Data are presented as mean ± SD. Groups sharing the same letter (a or b) are not statistically different from one another based on Tukey’s post hoc test. Each data point represents individual participant data and each group is represented by a different color. Dotted line indicates the proposed cutoff for an adverse postprandial triglyceride response. Abbreviations: NWL-Y—younger adults with normal-weight lean; NWL-O—older adults with normal-weight lean; NWO-O—older adults with normal-weight obesity; TG—triglycerides; HDL-C—high-density lipoprotein cholesterol.

**Figure 2:**
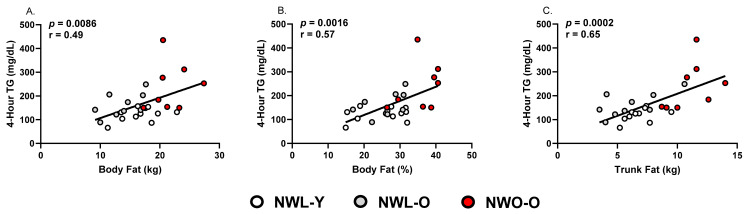
Correlations between body fat parameters and postprandial triglycerides. Pearson correlations between (**A**) absolute body fat and 4 h triglycerides, (**B**) body fat percent and 4 h triglycerides, (**C**) trunk fat and 4 h triglycerides. Each data point represents individual participant data. Abbreviations: NWL-Y—younger adults with normal-weight lean; NWL-O—older adults with normal-weight lean; NWO-O—older adults with normal-weight obesity; TG—triglycerides.

Individual triglyceride responses to the high-fat meal are visualized in Figure 1A. Fasting triglycerides were higher in NWO-O compared to NWL-Y (*p* < 0.05; η2 = 0.280; Figure 1B), though all groups were below the clinical cutoff of 150 mg/dL, on average. At the 4 h time point, NWO-O had ≥47% higher triglycerides than the other two study groups (*p* < 0.01; η2 = 0.317; Figure 1C). Mean 4 h triglycerides in NWO-O (239.4 ± 101.0) were also consistent with an adverse response based on an expert panel statement (i.e., ≥220 mg/dL; Figure 1C) [19]. Somewhat similarly, NWO-O had a higher absolute change in triglycerides versus NWL-Y (*p* < 0.01; η2 = 0.266; Figure 1D) and tended to have a higher change in triglycerides than NWL-O (*p* = 0.06; Figure 1D). Several body fat metrics were positively correlated with 4 h triglycerides in the full sample, including absolute body fat (*p* < 0.01; r = 0.49; Figure 2A), percent body fat (*p* < 0.01; r = 0.57; Figure 2B), and trunk fat (*p* < 0.01; r = 0.65; Figure 2C). NWL-O had higher fasting HDL-C than NWL-Y (*p* < 0.05; η2 = 0.284; Figure 1E) and had a greater decrease in HDL-C from baseline relative to both NWL-Y and NWO-O (*p* < 0.0001; η2 = 0.587; Figure 1G).

### 3.3. Fasting and Postprandial LBP, sCD14, and IL-6

**Figure 3:**
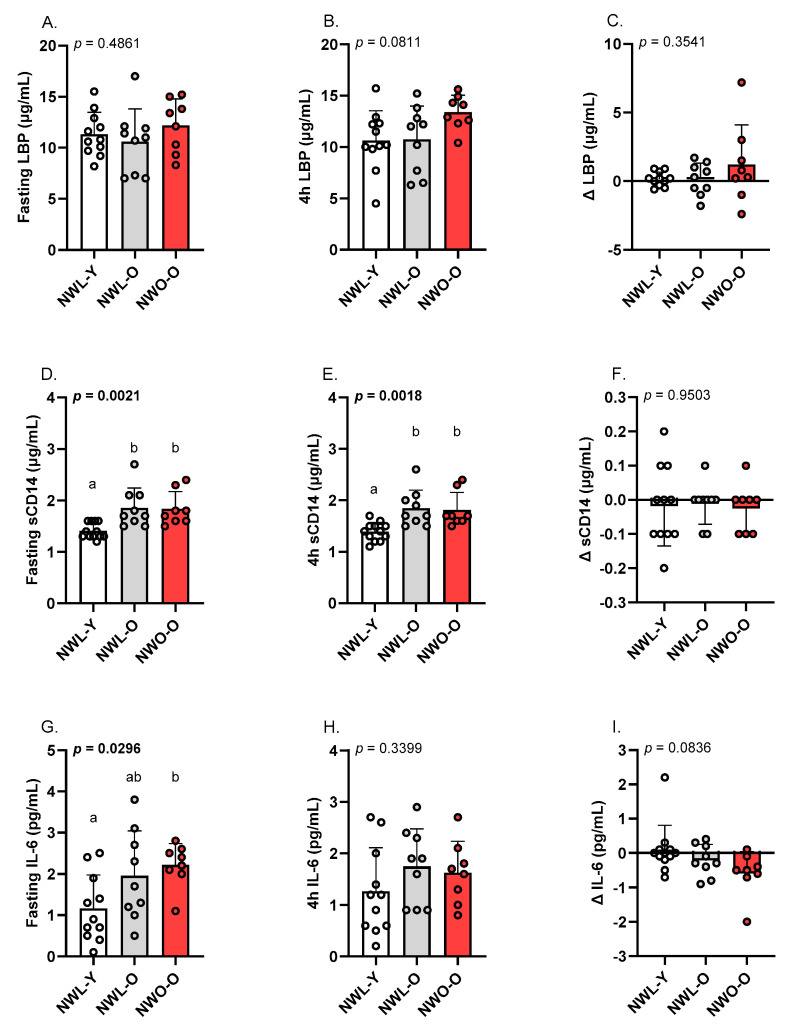
Fasting and postprandial LBP, sCD14, and IL-6: (**A**) fasting LBP, (**B**) 4 h LBP, (**C**) absolute change (∆) in LBP from baseline, (**D**) fasting sCD14, (**E**) 4 h sCD14, (**F**) absolute change (∆) in sCD14 from baseline, (**G**) fasting IL-6, (**H**) 4 h IL-6, (**I**) absolute change (∆) in IL-6 from baseline. Data are presented as mean ± SD. Groups sharing the same letter (a or b) are not statistically different from one another based on Tukey’s post hoc test. Each data point represents individual participant data. Abbreviations: NWL-Y—younger adults with normal-weight lean; NWL-O—older adults with normal-weight lean; NWO-O—older adults with normal-weight obesity; —lipopolysaccharide binding protein; sCD14—soluble cluster of differentiation 14; IL—interleukin.

Fasting and 4 h sCD14 were similarly higher in NWL-O and NWO-O relative to NWL-Y (*p*’s < 0.01; η2’s = 0.378–0.385; Figure 3D,E). However, the change in sCD14 following the high-fat meal was not different across groups (Figure 3F). Fasting IL-6 was higher in NWO-O compared to NWL-Y, while NWL-O was intermediate to these groups (*p* < 0.05; η2 = 0.245; Figure 3G). The 4 h and change in circulating IL-6 (Figure 3H,I), as well as baseline, 4 h, and Δ LBP (Figure 3A–C), were similar among study groups.

## 4. Discussion

The objective of this pilot study was to examine the acute cardiometabolic response to a high-fat meal in older adults with NWO. To accomplish this aim, we measured lipid parameters (i.e., triglycerides, HDL-C), biomarkers of intestinal permeability (i.e., LBP, sCD14), and the inflammatory marker IL-6 pre/post an oral fat tolerance test. To contextualize our data, we recruited younger and older adults with NWL in addition to the NWO-O group. Key findings include that NWO-O had higher 4 h triglycerides than both NWL-Y and NWL-O. However, when comparing the absolute change in triglycerides from baseline, NWO-O was only statistically different from NWL-Y. While not related to high-fat meal consumption per se, we also noted that fasting IL-6 was higher in NWO-O compared to NWL-Y, and fasting sCD14 was higher in both older adult groups relative to NWL-Y.

The triglyceride response following a high-fat meal challenge may be a sensitive tool to identify cardiometabolic risk in those with a largely normal clinical profile [15]. Mechanistically, elevated postprandial triglycerides are due to (1) sustained postprandial VLDL-triglyceride secretion secondary to hepatic steatosis and insulin resistance; (2) competition of these VLDL-triglycerides and diet-derived triglycerides within chylomicrons for clearance; and/or (3) impaired triglyceride clearance mechanisms (e.g., decreased lipoprotein lipase activity) [15]. In the present work, we documented that NWO-O had greater 4 h triglycerides than both NWL-Y and NWL-O. When examining the change in triglycerides from baseline, this pattern was largely the same. Importantly, we noted considerable variability in our NWO-O group’s 4 h triglyceride responses, suggesting metabolic heterogeneity in this population. While no other studies to our knowledge have assessed postprandial triglycerides in older adults with NWO, these data are similar to our previous work in middle-aged adults with NWO [11]. Specifically, middle-aged adults with NWO (mean age 34 years) had 66% higher 4 h triglycerides than a comparably aged NWL group.

Though the present study design prevents us from determining specific mechanisms driving higher postprandial triglycerides documented in NWO-O, our data point to several factors that should be examined in future studies. First, we noted that trunk fat (compared to absolute body fat and body fat percent) was most strongly correlated with 4 h triglycerides in our sample. These data imply that higher visceral fat—a major determinant of hepatic steatosis/hepatic insulin resistance and circulating VLDL-triglycerides within apoB particles—may be related to the greater postprandial triglyceride response in NWO-O [22]. Additionally, NWO-O engaged in less moderate-to-vigorous physical activity compared to NWL-Y. Previous work has identified that chronic exercisers have a lower postprandial triglyceride response than non-exercisers, which could be connected to our postprandial triglyceride data in NWO-O [15,23]. It should be noted that, beyond the fat content of our meal, its high glycemic load could have indirectly contributed to the postprandial triglyceride response through some combination of skeletal muscle insulin resistance and hepatic de novo lipogenesis. Taken together, it appears that NWO-O is characterized by altered postprandial triglyceride metabolism, but future work should confirm our initial observations and examine causal mechanisms.

Beyond adverse changes in circulating lipids, high-fat meals are known to impair the gut barrier [24]. This phenomenon, known as intestinal hyperpermeability, is conceptualized as a driver of cardiometabolic risk by promoting low-grade inflammation [24]. Indeed, several indicators of intestinal permeability (e.g., increased LPS, LBP, sCD14) are prospectively associated with CVD events, and greater postprandial endotoxemia is linked to development of type 2 diabetes [25,26]. For these reasons, we assessed fasting, 4 h, and change in LBP, sCD14, and IL-6. Interestingly, the high-fat meal did not impact markers of intestinal permeability and IL-6 in our study groups. These null findings could be related to our selection of a single, 4 h postprandial time point. We designed the present study to focus on the postprandial triglyceride response and to minimize participant burden. That stated, utilizing a single, 4 h postprandial time point reduced opportunities to observe changes in postprandial intestinal permeability and inflammation. However, examining our selected biomarkers at the 4 h time point was not completely inconsistent with previous literature. For example, a number of studies have reported peak IL-6 concentrations at 4 h after a high-fat meal [27,28,29,30]. However, it is not uncommon for peak IL-6 to be observed later in the postprandial period (e.g., 6 h) [31]. Similarly, changes in postprandial indicators of endotoxin exposure have been noted between two and four hours [16,32]. Nevertheless, we did document group differences in select parameters at individual time points. First, fasting IL-6 was higher in NWO-O relative to NWL-Y, but NWL-O was similar to both groups. These data are partially similar to data from Batsis et al. [10], who reported that C-reactive protein was higher in older males with NWO versus NWL counterparts. Second, we observed that fasting and 4 h sCD14 were increased in both NWL-O and NWO-O compared to NWL-Y. Given the change in sCD14 was +/−0.2 µg/mL across participants, it appears sCD14 was largely stable over the assessment window and not impacted by the high-fat meal. Previous research has identified that sCD14 increases with age [33], but we are unaware of other work documenting this change in older adults with normal BMIs specifically. In sum, our data supports the notion that NWO-O (and in some cases NWL-O) presents with basal low-grade inflammation. However, high-fat meal consumption did not affect measures of intestinal permeability and inflammation in our study. Future work should assess additional post-high-fat meal time points in NWO-O, consider additional biomarkers (e.g., direct LPS measurement), and consider whether gut microbiota composition is related to higher sCD14 in NWL-O and NWO-O

Our work possesses both limitations and strengths. First, we acknowledge our modest sample size. Though it was important to exclude individuals taking lipid-lowering medications to accurately assess postprandial triglycerides, this decreased our available pool of older adults to recruit from. As a result of our sample size, statistical power may have been insufficient to detect differences in certain participant characteristics (e.g., systolic blood pressure) and postprandial cardiometabolic factors (e.g., LBP). Further, our available degrees of freedom to include covariates in our analyses were limited due to sample size. Our overall sample was also skewed towards females, but sex distribution was similar across groups. Due to our decision to focus on the 4 h triglyceride response, it is also likely that we did not capture peak postprandial LBP, sCD14, and IL-6 as noted. On the other hand, including additional time points would have increased participant burden and may have impacted recruitment/feasibility. Nonetheless, future work should assess multiple postprandial time points. As another way to minimize participant burden, we only recorded diet and physical activity data for three days. However, these timeframes should be considered limitations given potential day-to-day step variability in older adults and possible underreporting of energy intake. We also acknowledge it would have been ideal to have measured postprandial glucose and insulin to examine their relationships with postprandial triglyceride data. Further, it is possible that our NWO-O group represents an example of “survivor bias.” In other words, NWO-O could be a subset of NWO individuals that reached older adulthood despite a high-risk phenotype, which should be considered when interpreting our data. Finally, it is worth noting that a limitation of the NWO literature is that many different definitions are utilized, making it challenging to compare studies directly in some cases.

A primary strength of this work is that it is among the first experimental studies in older adults with NWO. Additionally, we view inclusion of both younger and older NWL comparator groups as a strength of this study. Finally, our study design (i.e., test meal, time point selection) is based on current recommendations for postprandial triglyceride testing [15,19].

## 5. Conclusions

In sum, our data point to postprandial dyslipidemia as a potential mechanism contributing to CVD in older adults with NWO. This mechanism should be further examined in older adults with NWO, with the goal of improving CVD outcomes in this at-risk population. Moreover, both acute interventions (e.g., consumption of meals rich in mono- and poly-unsaturated fat) and chronic interventions (e.g., exercise/physical activity programs) should be studied with the goal of attenuating postprandial triglycerides in NWO-O. From a clinical implementation standpoint, our work points to the utility of (1) assessing body composition rather than just BMI, even in normal-weight individuals, and (2) the potential advantage of postprandial triglyceride testing in certain populations for uncovering cardiometabolic risk.

## Figures and Tables

**Table 1:** Participant characteristics. Data are presented as mean ± SD. Ranges for WC and WHtR are also presented. Omnibus *p*-values are displayed, and alpha was set at 0.05. Groups sharing the same letter (either a or b) are not statistically different from one another based on Tukey’s post hoc test. Abbreviations: NWL-Y—younger adults with normal-weight lean; NWL-O—older adults with normal-weight lean; NWO-O—older adults with normal-weight obesity; BP—blood pressure; BMI—body mass index; WC—waist circumference; WHtR—waist-to-height ratio; HDL-C—high-density lipoprotein cholesterol; LDL-C—low-density lipoprotein cholesterol; VLDL-C—very low-density lipoprotein cholesterol; ALT—alanine transaminase; AST—aspartate aminotransferase; HEI—healthy eating index; MVPA—moderate-to-vigorous physical activity.

Participant Characteristics	NWL-Y (n = 12)	NWL-O (n = 9)	NWO-O (n = 8)	*p*-Value
General/Body Composition				
Age (years)	21.0 ± 3.0 ^a^	71.0 ± 6.0 ^b^	67.0 ± 7.0 ^b^	<0.0001
Sex (M/F)	4/8	2/7	2/6	0.5514
Ethnicity (White/People of Color)	11/1	9/0	7/1	0.2950
Systolic BP (mmHg)	112.0 ± 13.0	124.0 ± 17.0	128.0 ± 17.0	0.0695
Diastolic BP (mmHg)	72.0 ± 8.0	75.0 ± 8.0	78.0 ± 5.0	0.2384
BMI (kg/m^2^)	22.5 ± 1.6	20.9 ± 2.1	22.8 ± 1.5	0.0671
WC (cm)	75.9 ± 6.1 (65.7–83.5)	77.5 ± 10.4 (67.9–97.8)	84.2 ± 7.4 (71.5–92.5)	0.0955
WHtR	0.43 ± 0.03 ^a^ (0.39–0.48)	0.48 ± 0.03 ^ab^ (0.42–0.58)	0.51 ± 0.04 ^b^ (0.45–0.56)	0.0023
Body Fat (%)	25.4 ± 6.6 ^a^	26.0 ± 5.2 ^a^	35.8 ± 5.3 ^b^	0.0013
Body Fat (kg)	17.0 ± 3.2 ^a^	13.7 ± 3.1 ^a^	21.8 ± 3.1 ^b^	<0.0001
Trunk fat (kg)	7.2 ± 1.4 ^b^	6.1 ± 2.3 ^b^	11.1 ± 1.8 ^a^	<0.0001
Lean Mass (%)	70.7 ± 6.5 ^a^	70.0 ± 4.9 ^a^	60.5 ± 5.3 ^b^	0.0013
Lean Mass (kg)	49.3 ± 11.9 ^a^	37.3 ± 7.8 ^b^	37.1 ± 5.5 ^b^	0.0072
Fasting Metabolic Parameters				
Glucose (mg/dL)	96.0 ± 4.0	102.0 ± 9.0	101.0 ± 11.0	0.1646
Total-C (mg/dL)	157.0 ± 24.0 ^a^	213.0 ± 22.0 ^b^	193.0 ± 27.0 ^b^	<0.0001
HDL-C (mg/dL)	60.0 ± 12.0 ^a^	79.0 ± 16.0 ^b^	64.0 ± 13.0 ^ab^	0.0183
LDL-C (mg/dL)	84.0 ± 16.0 ^a^	115.0 ± 20.0 ^b^	106.0 ± 19.0 ^b^	0.0034
Non-HDL-C (mg/dL)	97.0 ± 17.0 ^a^	133.0 ± 17.0 ^b^	129.0 ± 21.0 ^b^	0.0004
VLDL-C (mg/dL)	14.0 ± 3.0 ^a^	18.0 ± 5.0 ^ab^	22.0 ± 9.0 ^b^	0.0129
Triglycerides (mg/dL)	69.0 ± 16.0 ^a^	90.0 ± 24.0 ^ab^	107.0 ± 43.0 ^b^	0.0199
ALT (U/L)	27.0 ± 7.0	25.0 ± 7.0	22.0 ± 4.0	0.1760
AST (U/L)	26.0 ± 4.0	29.0 ± 5.0	27.0 ± 3.0	0.3203
Diet				
Energy (kcal)	2292 ± 419 ^a^	1744 ± 327 ^b^	1773 ± 557.4 ^ab^	0.0289
Carbohydrate (g)	270.1 ± 68.0 ^a^	197.4 ± 42.9 ^b^	207.1 ± 60.2 ^ab^	0.0345
Fiber (g)	22.5 ± 8.2	23.3 ± 6.9	21.7 ± 6.7	0.9151
Protein (g)	93.1 ± 31.1	79.1 ± 10.8	75.4 ± 13.4	0.2103
Fat (g)	94.7 ± 19.9	70.1 ± 25.0	73.9 ± 38.6	0.1796
Saturated fat (g)	29.5 ± 8.4	23.3 ± 8.4	22.7 ± 11.3	0.2751
Sodium (g)	3.7 ± 1.2	3.1 ± 1.1	3.3 ± 0.7	0.4597
HEI (0–100, a.u.)	52.0 ± 13.7 ^a^	64.1 ± 6.8 ^a^	63.5 ± 10.3 ^a^	0.0497
Physical Activity				
Steps/day	9338.0 ± 3207.0	8268.0 ± 3054.0	8417.0 ± 3456.0	0.7396
MVPA (min)	26.2 ± 36.6 ^a^	5.3 ± 10.7 ^ab^	3.3 ± 9.2 ^b^	0.0186

## Data Availability

Data are available from the corresponding author upon reasonable request.

## Supplementary Materials

The following supporting information can be downloaded at https://www.mdpi.com/article/10.3390/metabo15080550/s1, Table S1: Confidence intervals and effect sizes for study outcomes.
