# Supplementary material for: Postprandial Cardiometabolic Parameters in Older Adults with Normal-Weight Obesity: A Cross-Sectional Pilot Study
Source: Metabolites. 2025 Aug 15;15(8):550. doi: 10.3390/metabo15080550 (PMC12388539; doi:10.3390/metabo15080550)
Supplement: Supplementary file 1 [file metabolites-15-00550-s001.zip › metabolites-3767696-supplementary.pdf]

Outcome	NWL-Y 95% CI	NWL-O 95% CI	NWO-O 95% CI	Eta-Squared
Fasting TG	58.2 – 78.8	71.4 – 108.4	72.4 – 148.9	0.280
4-hour TG	103.0 – 164.9	124.4 – 200.7	154.9 – 323.9	0.317
Δ TG	35.3 – 95.5	33.9 – 111.4	71.4 – 193.6	0.266
Fasting HDL-C	51.8 – 67.2	64.2 – 94.4	52.8 – 75.2	0.284
4-hour HDL-C	49.5 – 65.2	56.2 – 84.6	46.7 – 73.0	0.154
Δ HDL-C	-3.8 – -0.5	-12.9 – -5.8	-4.7 – -1.3	0.587
Fasting LBP	9.87 – 12.8	8.1 – 13.1	10.0 – 12.4	0.056
4-hour LBP	8.7 – 12.6	8.2 – 13.2	12.0 –14.8	0.182
Δ LBP	-2.4 – 1.1	-0.8 – 1.0	-1.2 – 3.6	0.094
Fasting sCD14	1.3 – 1.5	1.6 – 2.2	1.6 – 2.1	0.378
4-hour sCD14	1.3 – 1.5	1.6 – 2.1	1.5 – 2.1	0.385
Δ sCD14	-0.1 – 0.1	-0.1 – 0.04	-0.1 – 0.03	0.004
Fasting IL-6	0.6 – 1.7	1.1 – 2.8	1.8 – 2.6	0.245
4-hour IL-6	0.7 – 1.8	1.2 – 2.3	1.2 – 1.8	0.083
Δ IL-6	-0.4 – 0.5	-1.1 – -0.1	-0.4 – 0.1	0.180

Table S1: Confidence intervals and effect sizes for study outcomes.
